# The impact of retirement on age related cognitive decline – a systematic review

**DOI:** 10.1186/s12877-017-0556-7

**Published:** 2017-07-21

**Authors:** Annette Meng, Mette Andersen Nexø, Vilhelm Borg

**Affiliations:** 1 The National Research Centre for the Working Environment, Lersø Parkallé 105, DK-2100 Copenhagen, Denmark; 2 The National Research Centre for the Working Environment, Steno Diabetes Center Copenhagen, Health Promotion, Niels Steensens Vej 6, DK-2820 Copenhagen, Gentofte Denmark

**Keywords:** Retirees, Cognitive functioning, Exit from labour market, Cognitive change

## Abstract

**Background:**

Knowledge on factors affecting the rate of cognitive decline and how to maintain cognitive functioning in old age becomes increasingly relevant. The purpose of the current study was to systematically review the evidence for the impact of retirement on cognitive functioning and on age related cognitive decline.

**Method:**

We conducted a systematic literature review, following the principles of the PRISMA statement, of longitudinal studies on the association between retirement and cognition.

**Results:**

Only seven studies fulfilled the inclusion criteria. We found weak evidence that retirement accelerates the rate of cognitive decline in crystallised abilities, but only for individuals retiring from jobs high in complexity with people. The evidence of the impact of retirement on the rate of decline in fluid cognitive abilities is conflicting.

**Conclusion:**

The review revealed a major knowledge gap in regards to the impact of retirement on cognitive decline. More knowledge on the association between retirement and age related cognitive decline as well as knowledge on the mechanisms behind these associations is needed.

**Electronic supplementary material:**

The online version of this article (doi:10.1186/s12877-017-0556-7) contains supplementary material, which is available to authorized users.

## Background

With the digitalisation of the society, cognitive functioning becomes more and more important for independent living in old age. The proportion of older adults in most Western countries is increasing [1, 2] and at the same time, ageing is generally associated with a decline in cognitive functioning [3, 4]. Therefore, knowledge on factors affecting the rate of cognitive decline and how to maintain cognitive functioning in old age becomes increasingly relevant.

The use it or lose it hypothesis proposes that our cognitive functioning deteriorates when we are not challenged or stimulated mentally. Accordingly, retirement can be expected to present a risk of accelerated cognitive decline due to a decrease in mentally challenging tasks following the exit from the labour market. However, the negative effect of retirement may differ between occupational groups. Individuals in occupations with high mental demands would be expected to show less age related cognitive decline while still in the workforce than individuals in occupations with low mental demands. According to the use it or lose it hypothesis, one would expect that the difference in rate of decline between these occupational groups would diminish after retirement, because their level of mental stimulation would become more similar. In other words, the negative effect of retirement can be expected to be greater for individuals retiring from jobs with high mental demands.

On the other hand, the theory of cognitive reserve See [5, 6] proposes that some individuals have a larger cognitive reserve than others. Two mechanisms behind this reserve are prosed: 1) *Brain reserve* which is a product of the brain anatomy so the larger brain and higher number of neurons and synapses an individual has, the larger the reserve. 2) *Cognitive reserve* reflects the extent to which an individual uses neural networks or cognitive paradigms efficiently and flexibly rather than anatomic differences [5, 6]. Cognitive reserve can be regarded as “the sum of its lifetime input” [7] (pp.617) and epidemiological studies suggest that educational level and occupational attainment can increase cognitive reserve [6, 8]. From this theoretical perspective, there are no differences in the *rate of decline* between individuals in occupations with high and low mental demands, only in *the level of* cognitive functioning. In other words, they show parallel trajectories of cognitive decline, also referred to as preserved differentiation. Accordingly, retirement would not affect the rate of cognitive decline in either of the two groups. However, individuals with a larger reserve would reach the level of clinical impairment at a later stage because of their generally higher level of cognitive functioning.

The trajectories of the cognitive decline depend on whether it is fluid or crystallised cognitive abilities. Fluid cognitive abilities include cognitive domains such as processing speed, working memory and spatial ability, while crystallised cognitive abilities include verbal ability and accumulated knowledge. Singer et al. [9] found fluid ability to decline with age while crystallised ability remained stable until the age of 90 after which they found evidence of decline. In addition, individual variance appears to increase with age for fluid but not crystallised cognitive ability [10, 11]. Based on this, it could be speculated that the potential detrimental effect of retirement will differ depending on whether it is fluid or crystallised abilities that are being measured.

Results from cross sectional studies suggest that there is a negative association between retirement and cognitive functioning [8, 12]. A limitation of cross sectional designs is that they are not able to provide evidence for a causal relationship. To overcome this problem, Rohwedder and Willis [13] used cross sectional data from various countries with different retirement ages. These aggregated data showed that drop in memory functioning matched the age of retirement in the respective countries. This pattern of results, they argue, provide evidence for a causal relationship between retirement and drop in memory functioning. However, the results from cross sectional designs only informs about differences in cognitive functioning, not changes in cognitive functioning over time because they only measure cognitive functioning at one point in time. It is therefore not possible to draw any conclusions on whether retirement affects the rate of cognitive decline based on results from studies using a cross sectional design. To answer this question, it is necessary to gather evidence from studies using a longitudinal design examining changes in cognitive functioning over time.

A review of longitudinal studies examining whether psychosocial work conditions can protect against cognitive decline did not find clear evidence that mental demands at work protect against cognitive decline (in neither crystallised nor fluid abilities) while still in the workforce [14], however, this study did not examine the impact of retirement on cognitive decline.

To the authors’ knowledge, no study so far has gathered the evidence from longitudinal studies on the impact of retirement on cognitive decline. Thus, a systematic overview of the knowledge on the impact of retirement on cognitive decline is still lacking.

Therefore, in the current article, we set out to answer the following research question: Does retirement affect age related cognitive decline? To answer this question we carried out a systematic literature review of longitudinal studies on the impact of retirement on cognition, gathering the evidence for the impact of retirement on cognitive functioning and/or the rate of change in cognitive functioning, assessing the evidence separately for fluid and crystallised cognitive abilities. In addition, we investigated if the association between retirement and cognitive functioning and/or rate of cognitive decline differed between occupations with different levels of cognitive demands.

## Methods

The systematic literature review followed the principles of the PRISMA statement [15].

### Search strategy

The literature search was carried out in four databases for articles in English published until the 1st of August, 2014:

1. Medline via the PubMed interface: http://www.ncbi.nlm.nih.gov/pubmed
2. PsycNET via the APA host interface: http://psycnet.apa.org/
3. Web of Science includes the three databases, *Science Citation Index Expanded (SCI-EXPANDED), Social Sciences Citation Index (SSCI) & Arts & Humanities Citation Index (A&HCI)* and was searched via the host interface: http://apps.webofknowledge.com/
4. OSH UPDATE includes the databases, CISDOC, HSELINE, NIOSHTIC, RILOSH, and was searched via the host interface: http://www.oshupdate.com/

The search string was defined by our inclusion/exclusion criteria and adapted to the interfaces of each database (the search string is available from the authors on request). Our inclusion and exclusion criteria are shown in Table 1.

**Table 1:** The inclusion and exclusion criteria

	Inclusion criteria	Exclusion criteria
Population	Employed or retired peopled aged 40+	Diseases, disorders, or medical conditions (e.g., brain diseases, dementia)
Design	Longitudinal studies: Observational cohort studies, case-control, or randomized controlled trials (at least 1 follow-up wave)	Cross-sectional studies, case studies, discussion papers, reviews, meta-analyses.
Exposures	Retirement Psychosocial working conditions e.g. mental job demands	Chemicals (e.g., solvents, manganese) physical demands, psychological distress
Outcomes	Age related cognitive decline and/or cognitive function	Outcomes that do not include a defined measurement of cognitive function (e.g., psychological health, psychological stress, depressive symptoms)

### Study evaluation

**Fig. 1:**
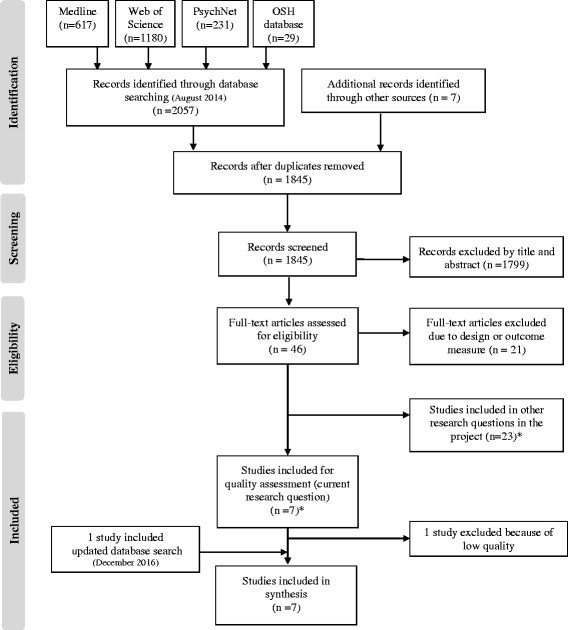
Flowchart illustrating the review process. *The total number of articles exceeds 25 because several of them relate to more than one of the research questions in the project

We evaluated the search results through three phases in the time period of August 2014 to June 2015. In the first phase titles and abstracts were screened according to the inclusion and exclusion criteria. In the second phase, the full texts of the potentially relevant studies were assessed according to the inclusion and exclusion criteria (See Table 1), and finally, we assessed the quality of the studies included. A flow chart of the review process is shown in Fig. 1.

#### Updated literature search

In December 2016 we completed an updated literature search following the procedure described above. We found 756 references out of which 754 were excluded by title and abstract and one was excluded after full text review. Thus, one additional study was included in the assessment.

#### Procedure for exclusion by tittle and abstract screening and by full text

Two researchers independently screened and categorised the titles and abstracts and later full text of the remaining articles based on the inclusion/exclusion criteria (See Table 1). The articles that were categorized differently by the two researchers were discussed at meetings with the senior researcher until a consensus was reached.

#### Quality assessment

The quality of all of the seven articles included was systematically assessed. All of the included studies were cohort studies with a longitudinal design. We therefore developed a quality assessment checklist based on existing guidelines for these types of studies based on strength and weaknesses of observational study designs [15–20]. We applied a scoring system of 0–10 points (0 indicating the poorest quality and 10 the highest quality). For each item on the checklist, the researcher assessed whether the study fulfilled the requirements and gave points accordingly (1 = yes, 0.5 = partially, 0 = no/information not available). We evaluated the transparency of the applied theory, aims, methods, results, and interpretation of the results, the quality of the available data sources and materials. We also evaluated whether the study design could overcome potential biases and confounders known to reduce the quality of cohort studies e.g., whether or not the study attended to drop-out and controlled for specific confounders relevant to the research hypothesis (See Additional file 1 for the quality assessment checklist).

The procedure for the consensus meetings regarding the quality assessment was the same as the procedure for the exclusion by full text described above.

Based on the score, each of the studies was categorised as high (8–10 points), moderate (6–7.5 points), low (4–5.5 points) or very low quality (0–3.5 points).

### Synthesis of strength of evidence

The quality of the studies was included in the evaluation of the strength of the evidence for each of the associations we examined:

1. *Strong evidence:* Consistent findings of minimum two studies of high quality.
2. *Moderate evidence:* Consistent findings from minimum two studies of minimum moderate quality or one study of high quality.
3. *Weak evidence:* Minimum one study of minimum moderate quality.
4. *Conflicting evidence*: Findings from at least one study of moderate or high quality that pointed in one direction and findings from a minimum of 33% of all studies of moderate or high quality that pointed in another direction.

## Results

A total of seven articles were included in the quality assessment. See Table 2 for an overview of the seven studies (an overview of the points given for each item on the quality checklist for each study is available from the authors on request).

**Table 2:** Overview of the seven studies included

Reference	Population	Exposure and confounders controlled for	Outcome	Follow up	Results
Andel et al. (2015) [21]	The US Health and Retirement Study (HRS). general population aged 55+ at baseline (*n* = 3779)	Self-reported retirement. Comparing the trajectories of cognitive change of participants with high and low job strain before and after retirement. Control for age, gender, education, marital status, race, income, length of occupation, depressive symptoms, cardiovascular disease, manual/non-manual work.	Episodic memory (immediate and delayed recall test). (F)^b^	10 data collection points at 2 years intervals. The participants averaged 7.4 interviews; 3.8 before retirement, 4.4 after retirement.	Growth curve model parameter estimates: Pre-retirement change was significant (−5.72, *p* > 0.01) Post-retirement change was not significant (0.92, *p* = 0.75) Greater job strain associated with worse episodic memory (−0.49, *p* < 0.001); not associated with pre-retirement change in episodic memory (0.39, *p* = 0.181); associated with greater decline in episodic memory post retirement (−0.65, *p* < 0.05).
Bonsang et al. (2012) [22]	The US Health and Retirement Study (HRS). General population aged 51–75 (*n* = 14,710)	Self-reported retirement (year and month when last employment ended). Compare retired with average score of sample. In the model they include eligibility for social security to provide evidence against reverse causality and they control for time in-variant heterogeneity.	Episodic memory (10 word recall test –immediate and delayed) (F) Working memory (subtract 7 from 100 up to five times). (F)	6 data collection points at 2 years intervals. Length of retirement included.	Retirement for one year or more has a negative effect on *episodic memory score* (Coefficient estimate: −0.942: 95%-confidence interval: −1.61 to −0.28). Magnitude of effect: −0.278, SE 0.100. *Working memory*: (coefficient estimate −0.279, SE 0.126). Magnitude of effect −0.230, SE 0.104 (*p* < 0.05).
Finkel et al. (2009) [29]	Swedish Adoption/twin study of Aging (SATSA). Twins aged 55+ (*n* = 462)	Self-reported year of retirement). Comparing slope of cognitive change before and after retirement, and comparing the trajectories of high and low complexity occupations. Control for dementia and practice effect.	Spatial ability (Figure Logic, Block design, Card Rotation) (F) Verbal ability (Information, Synonymous, Analogies) (C)^b^ Memory (Digit Span, Picture Memory, Names & Faces) (F) Processing speed (Symbol Digit and Figure Identification) (F)	5 measure points at 3 years intervals (one 7 years) Length of retirement measured (15 years before retirement to 20 years after retirement modelled).	Growth curve model parameter estimates: *Verbal ability*: Mean at retirement: (L)^a^: 53.7; (H)^a^: 55.3 (ns); pre-retirement change: (L): −0.13; (H): +0.07 (*p* < 0.05). Post retirement change: (L):-0.1: (H): −0.2 (ns). *Spatial ability*: Mean at retirement: (L): 51.36; (H): 54.36 *(p* < 0.05); pre-retirement change (L): −0.35, (H): −0.35 (ns); post retirement change (L): −0.29; (H): −0.51 (*p* < 0.05). *Memory:* So significant findings. *Processing speed*: Mean at retirement: (L): 52.93; (H): 55.47 (*p* < 0.05); pre-retirement change: (L): −0.27; (H): −0.52 (ns); post retirement change: (L): −0.60; (H): −0.69 (ns).
Fisher et al. (2014) [24]	US Health and Retirement Study (HRS). General population aged 51–61 at entry (*n* = 4182)	Self-report of year and month of retirement. Comparing rate of cognitive change before and after retirement, and comparing the trajectories of high and low mental demand occupations. Control for practice effect and socioeconomic, demographic, and health variables.	Episodic memory (immediate and delayed word recall test) (F) Mental status (telephone Interview of Cognitive Status)	Data from 1992 to 2010 collected at 2 years intervals. Participants included if completed min. 2 waves. Length of retirement included. (Mental status only post retirement measures)	*Episodic memory*: Difference in pre- and postretirement decline (Estimate = 0.06; *p* < 0.05). Higher mental demands associated with better memory (Estimate = 0.06; *p* < 0.05) and less steep decline (Estimate = 0.01; *p* < 0.05). *Mental status*: Higher mental demands associated with better cognitive status at point of retirement (Estimate = 0.11; *p* < 0.05). There was a general decline of 0.27 SD per 10 years post retirement. Higher mental work demands was associated with slower rate of decline (Estimate = 0.004; *p* < 0.05).
Roberts et al. (2010) [25]	The UK Whitehall II study. London-based civil servants aged 38–60 at entry (*n* = 2031)	Employment status. (still working vs fully retired at follow up, all working at baseline). Difference between baseline and follow up cognitive score comparing retirees and those still working. Control for adult IQ, age, mental and physical health, self-rated health, social class, education, psychosocial job characteristics, and leisure activities.	Short term verbal memory (free recall test) (F) Inductive reasoning (AH4 – part 1) (F) Verbal fluency (“s” words and animal names) (F)	5 years (159 had been retired between <1–115 weeks at follow up; 151 between 115 and 218 weeks; 160 between 219 and 309 weeks)	General trend of improved cognitive functioning at follow up. *Inductive reasoning*: retirees less improvement than those still working (Regression coefficient − 0.7; 95% CI −1.2 to −0.09). *Short term verbal memory*: no significant findings *Verbal fluency*: no significant findings
Wickrama et al. (2013) [26]	US HRS (Health and retirement study). General population aged 62+ at entry (*n* = 8524)	Self-reported work status: working full-time, working part-time, fully retired. They use structural equation models to investigate the reciprocal association between change in work status and cognitive change. Control for age, education, gender, race/ethnicity, depressive symptoms and physical disability.	Immediate memory (Recall from 10-word lists) (F)	6 data collection points at 2 years intervals. No information on length of retirement period.	Over three time intervals the level of working at one point in time predicted subsequent changes in immediate memory (β = .04, & .06, both *p* < 0.01, and β = .07, *p* < 0.001).
Ryan (2008) [27]	The Seattle Longitudinal Study (SLS). White middle and upper class individuals aged 60+ at entry (*n* = 271)	Work status (retired vs working). Comparing the cognitive scores of those working all three waves with those retiring during study. Control for gender, education, perceptual speed, subjective and objective health.	Verbal memory (PMA, immediate recall, delayed recall) (F) Inductive reasoning (PMA, ADEPT, word series, number series) (F) Verbal ability (PMA, ETS) (C)	3 data collection points at 7 years intervals. Length of retirement not measured.	*Inductive reasoning*: Participants employed in all three waves averaged 0.14 t-score units higher than those who worked only during one or two waves (*p* < 0.05). *Verbal ability:* Working more than one wave was associated with a 2.4 t-score unit gain (*p* < 0.01). *Verbal ability:* No significant findings

^a^ *H* high complexity with people jobs, *L* low complexity with people jobs. ^b^(*F*) Fluid cognitive ability, (*C*) Crystallised cognitive ability

All seven of the studies included measures of fluid cognitive abilities, two of the studies also included measures of crystallised cognitive abilities.

### Review of the seven studies that included measures of fluid cognitive abilities

Andel et al. [21] compared the trajectories of cognitive change of participants with high and low job strain respectively. They found that rate of cognitive decline before retirement was significant while the rate of cognitive decline after retirement was not, indicating a positive effect of retirement. In addition, they found that job strain was not associated with the rate of cognitive change before retirement, but after retirement, job strain was associated with greater decline in episodic memory, indicating that retirement has a less positive effect on participants who experience greater job strain. However, they did not directly assess the impact of retirement, so the results should be interpreted with caution. They control for socioeconomic, demographic, and health variables, but not for practice effect. We categorised the study as of high quality.

Bonsang et al. [22] compared the scores from the retired individuals with the average score of the sample and found that retirement was associated with an approximately 10% decrease in memory scores. However, they do not provide information on the impact of retirement on the rate of cognitive decline. We interpreted the effect size to be small. The study lacks information on drop out and it is therefore difficult to assess the risk of selection bias due to drop out. We categorised the study as moderate quality.

Finkel et al. [23] compared participants working in jobs with high and low complexity with people on cognitive performance and change in cognitive performance before and after retirement. They found that retirement was associated with an accelerated decline in processing speed for both job categories and an accelerated decline in spatial ability for participants retiring from high complexity jobs but not those retiring from low complexity jobs. They did not find any significant results for their third measure of fluid ability. We interpreted all effect sizes to be small. They also compared individuals retiring from jobs with high/low complexity with data and things, but did not find significant results. They control for practice effect and dementia. We assessed the study as being of moderate quality.

Fisher et al. [24] compared the results of participants retiring from jobs with high mental demands with participants retiring from jobs with lower mental demands on the rate of cognitive change before and after retirement. They found a decrease in memory score in the years leading up to retirement and a slightly less steep decline in memory score after retirement, indicating a positive effect of retirement on the rate of cognitive change. We interpreted the effect sizes to be small. Participants retiring from jobs with higher mental demands showed slightly higher performance and less steep decline both before and after retirement. Sample attrition may have affected the results. However, they control for socioeconomic, demographic, and health variables as well as practice effect. We categorised the study as of high quality in the quality assessment.

Roberts et al. [25] compared retired and those still working on the difference between the baseline score and the follow-up score. They found a general trend of improved cognitive functioning at follow-up. Participants who were retired generally showed less improvement than those still working, indicating a negative effect of retirement on the rate of cognitive change. However, this trend was only statistically significant for one out of their three measures of fluid ability and the effect sizes are small. They control for adult IQ, age, mental and physical health, self-rated health, social class, education, psychosocial job characteristics, and leisure activities. However, the results may have been biased both due to practice effect and drop outs. We rated the study as being of moderate quality.

Wickrama et al. [26] investigated the reciprocal association between change in work status and cognitive change. They found that work status predicted change in performance on the immediate memory test at follow-up sessions. Thus indicating that reduced working hours and retirement lead to decline in immediate memory. Again we interpreted the effect sizes as being small. They control for spurious findings due to common methods variance by including depressive symptoms and physical disability in their analyses. In addition, they control for age, education, gender, and race/ethnicity. However, they do not mention how they deal with drop outs or practice effect on the tests. It is therefore not possible to rule out that the results are biased due to these two factors. We categorised the study as of moderate quality.

Ryan [27] compared the cognitive scores of those working all three waves with those retiring during the study. She found a slight decline in performance for every increased year of age in all three cognitive domains she included. Retirement was associated with lower performance on one of the two measures of fluid ability. She did not directly assess the impact of retirement on the rate of decline, but from the results it appears that there is no difference in the rate of change between the groups. We interpreted the effect sizes to be small. She controls for gender, education, perceptual speed, subjective and objective health. The results can only be generalised to upper and middle class white Americans. T-tests show that drop outs had lower scores on verbal ability and processing speed, but there was no statistical significant difference on educational attainment, inductive reasoning or verbal memory. We categorised the study as of moderate quality.

### Synthesis of the evidence for the effect of retirement on the rate of change in fluid cognitive abilities

The evidence of the effect of retirement on rate of change in fluid cognitive abilities is conflicting.

One study of moderate quality [26] provides weak evidence of *a negative effect* of retirement on the rate of decline in fluid cognitive abilities indicating that retirement accelerates the rate of cognitive decline.

In addition, two studies of moderate quality have internally inconsistent results. They both get a mix of *not significant results* on some measures of fluid cognitive abilities and *a negative effect* of retirement on cognitive decline [23, 25] on other of their measures of fluid cognitive abilities.

Two studies of high quality [21, 24] provide strong evidence of *a positive effect* of retirement on rate of cognitive decline in fluid cognitive abilities indicating that retirement slows down the rate of cognitive decline. Individuals retiring from jobs with high mental demands [24] and individuals who experienced low job strain [21] in particular appear to experience a positive effect of retirement.

### Synthesis of the evidence for the effect of retirement on fluid cognitive functioning

One study of moderate quality [22] provides weak evidence of *a negative effect* of retirement on fluid cognitive functioning indicating that retirement leads to a drop in cognitive functioning.

In addition, one study of moderate quality has internally inconsistent results. It has a mix of *not significant results* on some measures of fluid cognitive abilities and *a negative effect* of retirement on cognitive functioning [27] on other of their measures of fluid cognitive abilities.

### Review of the two studies that included measures of crystallised cognitive abilities

Finkel et al. [23] (moderate quality) included one measure of crystallised abilities, verbal ability, and found a statistical significant difference in cognitive change before retirement where participants in high complexity jobs show an increase in ability while participants in low complexity jobs show a decrease. After retirement no statistical significant difference in cognitive change between participants in high and low complexity jobs was found, indicating that only participants in high complexity jobs experience a negative effect of retirement. We interpreted the effect sizes to be small. (Please find the more throughout description of the study under the review of studies including fluid abilities).

Ryan [27] (moderate quality) includes one measures of crystallised abilities, verbal ability, and found a slight decline in cognitive performance for every increased year of age. Retirement was associated with lower performance. She did not directly assess the impact of retirement on the rate of decline, but from the results it appears that there is no difference in the rate of change between the groups. We interpreted the effect sizes to be small. (Please find the more throughout description of the study under the review of studies including fluid abilities).

### Synthesis of the evidence for the effect of retirement on rate of change in crystallised cognitive abilities

One study of moderate quality [23] provides weak evidence for *a negative effect* of retirement on rate of decline in crystallised cognitive abilities indicating that retirement accelerates the rate of cognitive decline*,* but only for individuals retiring from jobs with high complexity with people.

### Synthesis of the evidence for the effect of retirement on crystallised cognitive functioning

One study of moderate quality [27] provides weak evidence of *a negative effect* of retirement *on crystallised cognitive functioning* indicating that retirement is associated with a drop in cognitive functioning among high SES individuals.

## Discussion

Regarding fluid cognitive abilities, the evidence for the impact of retirement was conflicting. We found strong evidence that retirement slows down and weak evidence that retirement accelerates *rate of cognitive change* as well as studies getting mixed results where they find that retirement has a negative impact on some measures and no impact on other measures of fluid cognitive abilities. In addition, we found weak evidence of a negative impact of retirement on *cognitive functioning* as well as a study getting mixed results of a negative impact of retirement on one measure and no impact on another measure of fluid cognitive abilities. Regarding crystallised cognitive abilities, we found weak evidence of a negative impact of retirement on both *rate of change* in cognitive functioning and *on cognitive functioning*.

The trajectories of the cognitive ageing depend on whether it is fluid or crystallised cognitive abilities [9]. However, as our results illustrate, there appears to be differences between domains within each of these two categories or at least fluid cognitive abilities. The match between job tasks and which cognitive measures to include in studies, may be of importance [28]. Following the use it or lose it hypothesis, different job types are likely to maintain different cognitive domains depending on which cognitive skills are needed to perform the job. Therefore retirement is likely to affect the various cognitive domains differently depending on which job the individual is retiring from. This could be one explanation for the conflicting evidence for the effect of retirement on age related decline in fluid cognitive abilities. Thus, the division of cognitive abilities solely into crystallised and fluid abilities may be too crude in this context and further division into cognitive domains within these two categories may be required. Given the small numbers of studies available for this review, further division into specific cognitive domains seemed inappropriate though. Nevertheless, in order to grasp the complexity of this issue, it may be informative if future studies take into account which cognitive domains are salient in the various job types included in the study and focus on these cognitive domains when measuring the impact of retirement.

In addition, we investigated differences in the impact of retirement on cognition between occupational groups with varying degrees of cognitive demands. On the one hand we found results indicating a less steep decline in fluid cognitive abilities as well as higher cognitive functioning post retirement in participants retiring from jobs with high mental demands compared with participants retiring from jobs with low mental demands [24] and also, that participants retiring from jobs with lower levels of job strain, mainly due to higher levels of control, experienced less cognitive decline post retirement than participants retiring from jobs with high level of job strain [21]. Andel et al., [21] point out that higher level of control may provide better options to develop and maintain behavioural strategies to cope with high demands and, thus, facilitate the development of more flexible neural networks. These findings suggest that cognitive reserve [5, 6] may actually have a protective effect on cognitive decline post retirement. However, the mechanism may not be the “exercising” of the brain per se as proposed by the use it or lose it hypothesis, but rather that certain types of jobs may provide more job control and thereby better conditions to develop neural compensation strategies. In addition, individuals retiring from jobs with high mental demands may largely consist of individuals who score high on the motivational trait “need for cognition”. The findings from Bear et al.[29] suggest that the motivational trait “need for cognition” has a protective effect on cognitive change post retirement. These individuals may not only be more attracted to jobs with high mental demands, but may also be more motivated to continue to challenge themselves mentally post retirement and this way, slow down the age related cognitive decline and reduce the negative impact of retirement. The mechanisms behind this protective effect could be the mental exercise per se as proposed by the use it or lose it hypothesis. However, again it could also be speculated that individuals have more control over how to perform cognitive tasks when retired and therefore, greater opportunity to develop neural compensation strategies and this way maintain a large cognitive reserve.

On the other hand, we also found results indicating that retirement accelerates cognitive decline to a larger extent among participants retiring from jobs high in complexity with people compared to participants retiring from jobs low in complexity with people, on measures of crystallised cognitive abilities and spatial ability (Fluid ability) (but this trend was not evident for processing speed or memory (both fluid abilities)) [23]. Finkel et al.[23] point out that they cannot rule out that psychological factors associated with retirement, such as depressive mood, are the cause of the accelerated decline in cognitive functioning found after retirement and that the results therefore may not necessary provide support for the use it or lose it hypothesis. They argue that retiring from a high complexity with people job may lead to greater negative psychological consequences because these workers may be more socially and psychologically attached to their job.

In short, the results indicate that the association between retirement and cognitive decline is affected by the characteristics of the job the person is retiring from. However, the mechanisms behind these associations are not yet clear. More research is needed to illuminate these associations and mechanisms, taking into account factors affecting cognition after retirement.

Our review revealed a major research gap. Because of the sparse number of studies and the heterogeneity of these studies, it was not possible to draw any firm conclusions on the impact of retirement on age related cognitive decline. In addition, data from four of these studies came from the same cohort study (US Health and Retirement Study). The PRISMA statement recommends removing duplicate publications to avoid bias [20]. However, three of the studies [21, 24, 26] used different predictor variables and we therefore regarded their results as independent. The fourth study [22] used a different outcome measure (cognitive functioning rather than rate of change in cognitive functioning) and was therefore not included in the same synthesis of evidence as the other three studies. We therefore included all four studies in our synthesis.

A great strength of our study is that it is the first study to complete a systematic review of longitudinal studies investigating the evidence for an impact of retirement on cognition, and distinguishing between cognitive functioning and cognitive decline as well as distinguishing between fluid and crystallised cognitive abilities. This stresses the salience of the knowledge gap revealed by our study.

## Conclusions

We only found weak and contradicting evidence for an association between retirement and age related cognitive decline. However, this systematic review revealed that there is a major research gap in this field. More knowledge on the association between retirement and age related cognitive decline as well as knowledge on the mechanisms behind these associations is needed. For example, how occupational characteristics influence the association between retirement and cognitive decline.

## Additional file

Additional file 1: Quality assessment check list. Description of data: The quality assessment list that was used in the evaluation of the studies. (DOCX 16 kb)
