# Supplementary material for: The impact of retirement on age related cognitive decline – a systematic review
Source: BMC Geriatr. 2017 Jul 21;17:160. doi: 10.1186/s12877-017-0556-7 (PMC5520232; doi:10.1186/s12877-017-0556-7)
Supplement: Additional file 1: — Quality assessment check list. Description of data: The quality assessment list that was used in the evaluation of the studies. (DOCX 16 kb) [file 12877_2017_556_MOESM1_ESM.docx]

**Appendix**

***Additional file 1: Quality assessment check list***

|  |
| --- |
| ***Questions to assess the quality of the way the information is reported***  *1. Did the study clearly describe mechanism/theoretical assumptions in relation to the aims? (0-1 point)*  *2. Did the study address a clearly focused issue? (0-1 point)*  *3. Was the design and method clearly described?*   - *# Exact number of participants at each follow up? (0-0.5* point)* - *# Information about differences between drop-outs and participants? (0-0.5* point)* |
| ***Questions to assess the quality of the available data sources***  *4. Was the cohort recruited in an acceptable way and in a way that minimized risk of selection bias? (0-1 point)*  *5. Was the participation rate acceptable?*   - *# Minimum 70 %? (0- 1 point)*   *6. Were the exposures and outcomes accurately measured to minimize measurement bias? (0-0.5* point)*   - *# Cognitive function measured by standardized cognitive tests (0-0.5* point)* |
| ***Questions to assess the quality of the way the available data sources are applied in the study***  *7. Was drop-out attended to by for example drop out analysis or sensitivity analysis?( 0-1 point)*  *8. Inclusion of appropriate confounders?*   - *# Educational level/socioeconomic status (0-0.5* point)* - *# Pathology (0-0.5* point)* |
| ***Questions to assess the applicability of the results***  *9. Was it possible to interpret the results without bias or confounders?; applicability of results considering strengths and limitations (0-1 Point)*  *10. Were the results plausible according to the aims, theoretical mechanisms, study context and previous literature? (0-1 Point)*   - *Strength of association according to statistical power and significance levels/confidence intervals* - *Bradford Hills criteria (time sequence, dose-response gradient, plausibility, consistency)* |
| ***Total points (0-10)***: |

Rating of the answers to the questions: Yes=1 point; partially=0.5 point; no/information not available=0 points

* Yes only gives 0.5 point because it is a sub-question.

# means the criteria must be fulfilled to get points
